# A qualitative inquiry of access to and quality of primary healthcare in seven communities in East and West Africa (SevenCEWA): perspectives of stakeholders, healthcare providers and users

**DOI:** 10.1186/s12875-021-01394-z

**Published:** 2021-02-25

**Authors:** Soter Ameh, Bolarinwa Oladimeji Akeem, Caleb Ochimana, Abayomi Olabayo Oluwasanu, Shukri F. Mohamed, Samson Okello, Alfa Muhihi, Goodarz Danaei

**Affiliations:** 1 Department of Community Medicine, Faculty of Medicine, College of Medical Sciences, University of Calabar, Calabar, Cross River State Nigeria; 2 Lown Scholars Program, Department of Global Health and Population, Harvard T.H. Chan School of Public Health, Boston, MA USA; 3 Department of Epidemiology and Community Health, University of Ilorin, Ilorin, Nigeria; 4 Ochimana Caleb Foundation, Federal Capital Territory, Abuja, Nigeria; 5 University Health Services, University of Ibadan, Ibadan, Nigeria; 6 Health and Systems for Health Unit, African Population and Health Research Center (APHRC), Nairobi, Kenya; 7 Department of Internal Medicine, Mbarara University of Science and Technology, Mbarara, Uganda; 8 Division of Infectious Diseases and International Health, Department of Medicine, University of Virginia Health Systems, Charlottesville, VA USA; 9 Africa Academy for Public Health, Dar es Salaam, Tanzania; 10 Department of Community Health, Muhimbili University of Health and Allied Sciences, Dar es Salaam, Tanzania; 11 Department of Epidemiology, Harvard T.H. Chan School of Public Health, Boston, MA USA

**Keywords:** Access, Quality, Primary healthcare, Universal health coverage, Health-seeking behaviour, Social entrepreneurship, Nigeria, Kenya, Uganda, Tanzania

## Abstract

**Background:**

Universal health coverage is one of the Sustainable Development Goal targets known to improve population health and reduce financial burden. There is little qualitative data on access to and quality of primary healthcare in East and West Africa. The aim of this study was to describe the viewpoints of healthcare users, healthcare providers and other stakeholders on health-seeking behaviour, access to and quality of healthcare in seven communities in East and West Africa.

**Methods:**

A qualitative study was conducted in four communities in Nigeria and one community each in Kenya, Uganda and Tanzania in 2018. Purposive sampling was used to recruit: 155 respondents (mostly healthcare users) for 24 focus group discussions, 25 healthcare users, healthcare providers and stakeholders for in-depth interviews and 11 healthcare providers and stakeholders for key informant interviews. The conceptual framework in this study combined elements of the Health Belief Model, Health Care Utilisation Model, four ‘As’ of access to care, and pathway model to better understand the a priori themes on access to and quality of primary healthcare as well as health-seeking behaviours of the study respondents. A content analysis of the data was done using MAXQDA 2018 qualitative software to identify these a priori themes and emerging themes.

**Results:**

Access to primary healthcare in the seven communities was limited, especially use of health insurance. Quality of care was perceived to be unacceptable in public facilities whereas cost of care was unaffordable in private facilities. Health providers and users as well as stakeholders highlighted shortage of equipment, frequent drug stock-outs and long waiting times as major issues, but had varying opinions on satisfaction with care. Use of herbal medicines and other traditional treatments delayed or deterred seeking modern healthcare in the Nigerian sites.

**Conclusions:**

There was a substantial gap in primary healthcare coverage and quality in the selected communities in rural and urban East and West Africa. Alternative models of healthcare delivery that address social and health inequities, through affordable health insurance, can be used to fill this gap and facilitate achieving universal health coverage.

**Supplementary Information:**

The online version contains supplementary material available at 10.1186/s12875-021-01394-z.

## Background

In 2008, the World Health Organization (WHO) reported that people were healthier and lived longer than in 1978 when the Alma Ata Declaration was signed [1]; hence, affirming the progress made toward achieving “health for all”. Some countries, especially those that make up today’s middle-income countries, made great improvements and were on track to achieve the targets in the health-related Millennium Development Goals. In Chile, Malaysia, Portugal and Thailand, mortality rates were less than one-fifth of what they were in 1978 [1]. On the other hand, 20 of the 25 countries where under-five mortality was two-thirds or more of the 1978 levels were in sub-Saharan Africa (SSA) [1]. The high levels of mortality in SSA were associated with poor access to healthcare [1].

Nearly a decade after the WHO proposed Primary Health Care (PHC) reform to refocus health systems toward health for all, specifically universal coverage reform to improve health equity [1], at least one-half of the world’s population still lack access to essential health services [2]. There is evidence of severely limited access to primary healthcare in SSA [3, 4]. This is primarily due to inability of households to pay user charges as most health systems in Africa depend heavily on out-of-pocket payments by users [5]. In extreme cases, a large section of the population in SSA have been impoverished by healthcare payments or forgo treatment due to inability to defray health bills [6]. Hence, inequities in health financing remains a major challenge in SSA [7]. The implication of lack of financial access to healthcare is the proliferation and utilisation of patent medicine vendors (PMVs) [8–10], defined as persons without formal pharmacy training who sell orthodox pharmaceutical products on a retail basis for profit [11]. The choice of utilisation of PMVs has been attributed to geographical accessibility, shorter waiting times, more reliable drug stocks, longer opening hours, greater confidentiality, more personable social interactions, ease of seeking advice, low cost of services and flexible pricing system, and no separate fee charged for consultation [12].

A key implication of inability of countries in SSA to deliver adequate quality healthcare is health workforce migration [13]. Migrant doctors and nurses in SSA do not wish to take up available posts in primary and first-contact care in their home countries due to poor working environment; difficult living experiences; poor career path; lack of basic equipment and medicines; work overload; lack of professional support; security concerns; lack of opportunities for education of children, especially in rural areas; poor remuneration and the perception that primary care is of a lower status that hospital medicine [14–17]. Furthermore, poor-quality healthcare is associated with excessive mortality. Of the 8.6 million excess deaths that were amenable to healthcare in 2016 globally, 5.0 million is attributable to poor-quality healthcare [18].

There is a renewed effort to address health inequity through Universal Health Coverage (UHC) which would result in all people accessing quality essential health services without exposure to financial hardship. The WHO and World Bank have acknowledged, within the context of the Sustainable Development Goals (SDGs), UHC as a means to improve population health and reduce the financial burden of healthcare in LMICs. Of the 17 SDGs adopted by the United Nations General Assembly in September 2015, SDG 3 focuses on ensuring healthy lives and promoting well-being for all at all ages. Target 3.8 of SDG 3 – achieving UHC, including financial risk protection, access to quality essential healthcare services and access to safe, effective, quality and affordable essential medicines and vaccines for all – is the key to attaining SDG 3 as well as the health-related targets of other SDGs, [2, 19].

We, therefore, conducted a multi-site study to provide in-depth viewpoints of healthcare users, healthcare providers and stakeholders such as leaders of landlord associations, community-based organisations and traditional/religious leaders regarding access to and quality of healthcare in four communities in Nigeria, West Africa and one community each in three East African countries - Kenya, Uganda and Tanzania.

## Methods

### Study settings

This was a community-based study conducted in four Nigerian communities (Okpok Ikpa, Ikire, Ogane-Uge, and Olorunda Abaa) and one community each in Kenya (Viwandani), Uganda (Soroti), and Tanzania (Ukonga). The communities were chosen based on existing collaborations with investigators in each site. The co-authors (SA, BOA, CO, AOO, SFM, SO and AM) are Visiting Scholars/Scientists at the Lown Scholars Program in Cardiovascular Health, Department of Global and Health Population, Harvard. T.H. Chan School of Public Health, Harvard University, Boston, USA. GD is a professor in the same department and the coordinator of the program. The focus of the collaboration is to set up Lown Community Health Centres (LCHCs) in the study sites and run them as a social enterprise. But this had to be preceded by conducting a community (quantitative and qualitative) survey/diagnosis in these sites. The focus of the qualitative study was to identify gaps in access to and quality of primary healthcare as well as health-seeking behaviour of community members. It is intended that evidence generated from this study will be used to inform setting up the LCHCs. This paper, which explores the deductive themes on access to and quality of healthcare as well as health-seeking behaviour, is an output of the qualitative component of the community survey. Additional file 1 shows the profile of the eight communities where this study was conducted.

### Study design and population

This was a thematic analysis of health-seeking behaviour, access to and quality of primary healthcare in healthcare facilities and other health-related institutions in seven communities across the four African countries. The study population consisted of healthcare users [young people (25–44 years of age), middle-aged people (45–60 years of age) and elderly people (60–75 years of age)] and healthcare providers. The WHO’s classification of age periods of human life was adopted in selecting the study population [20]. Other study population included stakeholders such as traditional/religious leaders and leaders of landlord associations and community-based organisations. The rationale for selecting these respondents was to get a balanced and in-depth perspective of lived experiences with access to and quality of care in health facilities in the study settings.

### Inclusion and exclusion criteria for research participants

Inclusion criteria were adults 18 years and above and community members residing in the communities for one or more years. Individuals judged to not have autonomy of decision making were excluded.

### Conceptual framework

**Fig. 1:**
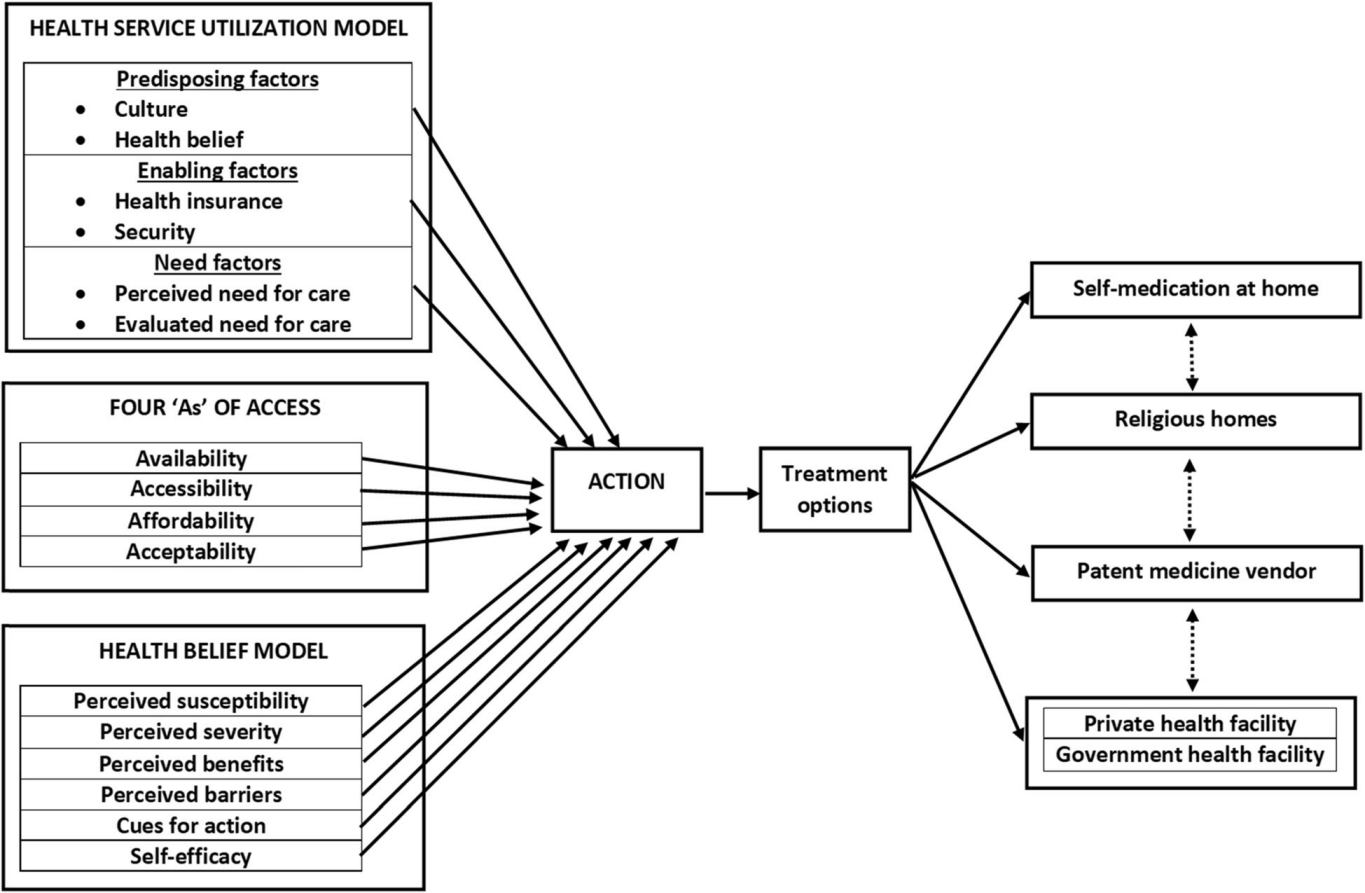
Conceptual framework for the study

A conceptual framework (Fig. 1) was developed to facilitate the exploration and interpretation of the themes on access to and quality of primary healthcare as well as health-seeking behaviour. As was used elsewhere, [21] this conceptual framework combined the elements of the Health Belief (HB) Model, [22] Health Care Utilisation (HCU) Model, [23] the four ‘As’ of access to care (Availability, Accessibility, Affordability, and Acceptability), and the pathway model [24] to better understand these themes from the perspectives of healthcare providers and users as well as other stakeholders from diverse cultures, ethnicities and religious backgrounds across the four countries.

The HB Model indicates that treatment options depend on perceptions of susceptibility to and severity of illness, perceived benefit of and barriers to care, cues to action (promoting awareness), and self-efficacy (providing guidance) [22]. The purpose of the HCU Model is to identify conditions that either facilitate or impede healthcare utilisation. According to the HCU Model, response to ill-health depends on predisposing factors (existing culture and health beliefs), enabling factors (e.g. health insurance) and need factors such as perceived and evaluated need for medical care [23]. Concerning the fours ‘As’ of access, people take action to address their ill-health if health services are available, accessible, affordable and acceptable [24]. The double-headed dotted arrows indicate the pattern in which participants switched from one treatment option to another depending on their satisfaction with or response to treatment received.

### Data collection

The interviews were conducted in all the study sites from June to October 2018. Table 1 shows the research format used in the research sites. The site lead researchers determined an appropriate method for collecting the data based on site-dependent peculiarities. Twenty four (24) focus group discussions (FGDs, *n* = 155) were held for healthcare users (young, middle-aged and elderly men and women), 25 in-depth interviews (IDIs) were conducted with healthcare providers and village leaders (healthcare providers and users participated in the IDIs in Kenya) and 11 key informant interviews (KIIs) were conducted with stakeholders. Prospective participants were selected using purposive sampling methods (Additional file 2). Participants were interviewed in the local languages (Efik, Yoruba, Igala and Swahili) in a private location within their communities. The FGDs lasted 60–90 min and were held for 6–11 participants of similar age range. The IDIs and KIIs lasted about 30 min. The interview guides were developed for this study and contained the three study themes on access to and quality of healthcare as well as health-seeking behaviour (Additional file 3).

**Table 1:** Qualitative research format used in the study sites in 2018

Study site	Number of FGDs	Number of KIIs	Number of IDIs
Okpok Ikpa, Nigeria	8	0	1
Ikire, Nigeria	4	0	0
Olorunda, Nigeria	4	2	0
Ogane-Uge, Nigeria	2	0	1
Viwandani, Kenya	4	9	7
Soroti, Uganda	0	0	14
Ukonga, Tanzania	2	0	2
Total	24	11	25

### Quality assurance

The qualitative fieldworkers with 5–10 years field experience received training for two to three days. Community mobilisers guided the fieldworkers in a few study sites while field guides were recruited to guide the fieldworkers in other sites that did not have community mobilisers. One field worker audiotaped and moderated the discussions while another took notes and documented non-verbal cues. The interview guides, originally written in English, were translated into local languages. Audio recordings were translated and transcribed into English. The site lead researchers (SA, BOA, CO, AOO, SFM, SO and AM) listened to the audio recordings to identify errors and discrepancies.

For the purpose of trustworthiness of the data collected and the qualitative research process used, concerns on credibility, transferability, dependability and reliability were addressed. To enhance credibility, the data were triangulated by administering questions on the three themes in the topic guides to categories of participants as was explained under the data collection sub-section above.

The qualitative research process is described to enable transferability of findings of this research to other settings. For example, a description of the study settings, existing collaborations between the co-authors, context in which the research was carried out, study sample (191 purposively sampled respondents), and inclusion/exclusion criteria [25]. Furthermore, the purpose of conducting the FGDs, IDIs and KIIs with respondents was to enable an in-depth exploration of lived experiences with health-seeking behaviour, access to and quality of healthcare, capitalising on group interactions. The FGDs were held in designated venues in the communities to encourage participants to freely express and communicate their experiences.

Dependability and reliability of the data were enhanced through coding and verification of inconsistent codes as presented under the Data Analysis sub-section below. Overall, 24 FGDs, 25 IDIs and 11 KIIs were conducted and more could not be done because data saturation was achieved [26].

### Data analysis

We conducted a thematic analysis of transcribed FGDs and interviews using the MAXQDA 2018. A combination of deductive and inductive analytical approaches was used. Deductive analysis was based on three themes identified a-priori: access to healthcare, quality of healthcare and health-seeking behaviour. Emerging themes, patronage of patent medicine vendors and quackery by patent medicine vendors (PMVs - drug vendors who provide the first and the main point of care in communities), that were not identified a-priori were analysed inductively.

The corresponding author, SA, coded the data which were verified by the co-authors (SA, BOA, CO, AOO, SFM, SO, AM and GD) through the reading and re-reading of the quotes. A code book was developed based on recurring pre-identified themes and emerging themes.

The analytical process began with multiple readings of the transcripts and coding of the data through an iterative process. A codebook (a list of codes) was developed based on the recurring themes and subthemes. Interesting features in the texts were highlighted and dragged to the generated codes that reflect the meanings in the segments of the transcript. Each code and its texts were assigned the same colour to distinguish one code from another. Thereafter, some codes were grouped together in a hierarchical manner, like a coding tree, to form an overarching theme. Reliability of the coded data was verified through discussions of inconsistent codes among co-authors until agreement was reached.

## Results

The main findings showed limited access to healthcare and unacceptable healthcare quality. Having health insurance was an enabling factor in utilising healthcare in East Africa while use of herbal medicines and other traditional medicine practices delayed or deterred seeking modern healthcare. Healthcare providers, healthcare users and stakeholders mentioned insufficient equipment, frequent drug stock-outs and long waiting times as major quality issues in the provision of quality health services. On the other hand, the respondents differed in their opinions on general satisfaction with quality of care with healthcare providers reporting good quality care in the same sites where healthcare users reported poor quality care.

### Access to primary healthcare

#### Facilitators

##### Free health services

Provision of free health services by some public facilities was perceived by respondents to facilitate access to primary healthcare by providing free drugs and tests, but this was often associated with long waiting time. On the other hand, private facilities were thought to be unaffordable to many users even though waiting time was shorter.

*What I like most about the government hospital is the fact that you don’t pay. If you are lucky and you find when drugs are available, you will get drugs. And even testing will be done for free and drugs you get for free and even if its HIV, they test you for free [IDI with an elderly man, Uganda].*

#### Health insurance

A respondent whose opinion reflected most other participants’ perception was that having a health insurance was a facilitator of access to primary healthcare irrespective of the cost of treatment.

*It (referring to a type of health insurance scheme) caters for all health services regardless of the illness. It doesn’t categorize if its amputation or laboratory services [FGD Respondent 1 (young man), Kenya].*

However, some respondents had varying opinions regarding inability to access certain services which necessitated out-of-pocket expenditures in certain health facilities that did not accept health insurance cards from some insurance providers.

*X health insurance scheme helps very little because they have to pay for some services in cash. Sometimes people have to dig into their pockets. It doesn’t cover everything because there are some drugs you’ll be told to buy [KII with a religious leader, Kenya].*

*Again the cards that ought to be used (participant mentions names of some insurance providers) are supposed to be accepted and used for outpatient services. They are not used for accessing services (KII with a community-based organisation leader, Kenya).*

*I went to X dispensary with my insurance card, I was rejected (everyone laughs) [FGD Respondent 1 (elderly woman), Tanzania].*

### Barriers

Respondents mentioned that high costs of care, long distance to health facilities, unavailability or shortage of health personnel, limited operating hours of healthcare facilities and patronage of PMVs were barriers to accessing healthcare.

#### Out-of-pocket payments and inability to afford high costs of care

Some respondents reported having challenges in accessing health services due to exorbitant fee-for-service charged in private facilities.

*The last time I went to X health facility, something entered my ear. They gave me a letter and sent me to another health facility, but I didn’t have money. So I tore the letter and stayed with that thing inside my ear [FGD Respondent 3 (young woman), Kenya].*

*You also pay for investigations. Nothing is for free. You have to pay for everything. For us who are poor, we face a lot of challenges getting medical services [FGD Respondent 6 (young woman), Tanzania].*

*When I asked them to attend to me first since I was bleeding they asked me for money which I didn’t have then. I was forced to deposit my phone after which they attended to me. So I had to go sort the finances out and come later to collect it. [FGD Respondent 2 (young man), Kenya].*

#### Distance to health facilities

Far distance to healthcare facilities posed a challenge to respondents. The cost of seeking healthcare was further increased by transportation costs.

*Most people trek, like those without a car or a motorcycle. Once you are sick and you stand by the road and there is no bike to take you there (referring to the health facility), you have to walk there. Like where I live, I trek o! (o is an exclamation in Nigeria used to emphasis or buttress an argument). It takes me like one hour twenty something minutes [FGD Respondent 1 (middle-aged woman), Okpok Ikpa, Nigeria].*

#### Unavailability of health personnel

Respondents commonly reported unavailability of doctors and nurses in health facilities.

*You know at times the doctors and nurses have their other private clinics. So they leave the other side of the government and go to the other side of the private. So when you go there, you may not get them easily [IDI with a young man, Uganda].*

*They don’t even have the capacity to handle malaria. They only have a Community Health Officer and there is no competent nurse there [FGD Respondent 2 (young man), Olorunda, Nigeria].*

*I went there the first time I didn’t get the doctor, the second time I didn’t get the doctor. It was the third time that I received the services I needed [FGD Respondent 4 (young woman), Kenya].*

*The last time I visited there, the District Health Assistant-in-charge was not available. I had to travel to another town to buy drugs from a patent medicine store [FGD Respondent 2 (middle-aged women), Ogane-Uge, Nigeria].*

#### Health service days and hours

Most respondents reported public facilities did not operate for 24 h during the day and during weekends, unlike in private facilities.

*Many times I had wanted to go to voluntary counselling and testing, but my husband was at work (during weekdays). If you go to facility A, you find that the hospital is closed over the weekend and that is when my husband has the time (Sunday afternoon) to visit the clinic. So it’s a real problem [FGD Respondent 3 (young woman), Kenya].*

*For example, in Facility B, if opening time is 9:00am, if you go there at 12:00 pm, they will not serve you because the time is gone [FGD Respondent 7 (elderly woman), Kenya].*

### Quality of primary healthcare

#### General (dis) satisfaction with quality of care

There was a variety of responses regarding quality of care. While some respondents reported satisfaction with quality of care, others reported dissatisfaction.

*We are not condemning the health facility (referring to a specific health facility). I remember a team of white people came here and I was operated upon. I enjoyed it very well (referring to the services) [FGD Respondent 6 (middle-aged man), Okpok Ikpa, Nigeria].*

*I think the quality is good (referring to a private facility). I can give (referring to satisfaction rating) them good [IDI with an elderly man, Uganda].*

*You can meet a doctor or nurse and he will not listen to you (referring to a public facility). While you are still explaining how you feel, even before you finish, they write you prescription to get medicine (mmh). Now you keep asking yourself what is this medicine for [FGD Respondent 3 (young woman), Tanzania)?*

#### Health personnel factors

Retention of qualified health personnel in public facilities was reported to be challenging due to low remuneration. Furthermore, health personnel working in public facilities were said to be sometimes unprofessional toward their clients. On the other hand, respondents reported that health personnel in private facilities were unqualified and lacked training.

*We try as much as possible to employ qualified medical personnel for high standards in the provision of services. For remuneration, we don’t pay them what they expect to be paid, so sometimes we lose staff. So staff turnover is higher than what we desire [KII with a healthcare provider in a private facility, Kenya].*

*Even those ones (referring to private facilities) don’t have qualified staff. They are not trained. Some people who have worked in pharmaceutical shops are just recruited and they just dress them up to attend to people [FGD Respondent 1 (elderly man), Olorunda, Nigeria].*

*But again government employees don’t treat people well. You can ask them a good question and they answer you with an attitude. So, if you have money, you better go to private hospitals or to chemist [FGD Respondent 1 (young woman), Kenya].*

*Hmmm, at times nurses tend to be rude [IDI with a young woman, Uganda].*

#### Waiting time

Respondents reported that waiting time was longer in public health facilities due to late arrival of doctors. Long queues in these facilities were said to be the reason why people visited the PMVs.

*You don’t take time (referring to a short waiting time in a private facility) unlike the main hospital (referring to a long waiting time in a public facility) where you go in the morning and it takes almost the whole day for you to be fully attended to because of the population [IDI with an elderly man, Uganda].*

*If you go to the General Hospital, just have it at the back of your mind that it is when God releases you that you will leave the place. By now (referring to early in the morning), people will already be many there and the doctor will not come until 11.00 am because government work is not the work that you will be sweating over [FGD Respondent 5 (elderly woman), Ikire, Nigeria].*

*So, it (referring to long waiting time) forces you to go to the chemist no matter the cost since in the public facility, there is a long queue [FGD Respondent 2 (young man), Kenya].*

#### Drug stock-outs

Some respondents reported that healthcare was free in public facilities, but drugs were frequently out-of-stock; hence, forcing patients to go to PMVs.

*Even after the doctor sees the person, he just hands him a prescription to buy his drugs outside the hospital and as such, people buy the quantity they can afford and not the complete dose [FGD Respondent 3 (young man), Ikire, Nigeria].*

*The city council has indicated that the health services are free and when you seek help, they prescribe drugs. But upon going to retrieve them, you are told they have none [FGD Respondent 6 (elderly man), Kenya].*

*There were no drugs in the clinic, and even common detergent was not available. I regretted going there [FGD Respondent 2 (young woman), Ogane-Uge, Nigeria].*

#### Interrupted power supply

Erratic electricity supply was reported by several respondents in Nigeria.

*It is very difficult to get medical tests done in this community, even at the public hospital. I have to buy three litres of fuel (referring to the purchase of petrol to power the hospital-owned electric generator, which is the responsibility of the hospital) each time I want to have a blood test done as ordered by the doctor. I have done this six times over the last two months [FGD Respondent 1 (elderly man), Ikire, Nigeria].*

#### Lack of equipment

The lack of equipment used to conduct basic tests was reported to negatively impact the quality of service delivery.

*Again service delivery is a challenge since they (referring to public facility) lack equipment to carry out tests [FGD Participant 5 (young man), Kenya].*

### Health-seeking behaviour

Most respondents reported that their first action during a minor illness was self-treatment at home with local herbs, often administered through enema, or medicines previously bought from PMVs. This practice was widely reported in the Nigerian sites where there is a traditional belief that enema purges the body of impurities. The next line of action was to visit a PMV. If medicines bought from a PMV was not effective, respondents would then seek treatment in health facility.

#### Self-medication at home

Self-medication with herbs and drugs bought from chemists was reported to be a common practice to treat illness at home.

*I pump o (referring to self-administration of herbs through the anus using a pump-like device) because in this our community, we believe in tradition. Once you are sick, the first thing that you would do is to wash your system out before treating. We have leaves and herbs that can help us in this community to wash our system [FGD Respondent 2 (middle-aged woman), Okpok Ikpa, Nigeria].*

*Sometimes I use Aloe Vera when I feel I have malaria (mmh). We also use it well and feel better [FGD Respondent 4 (elderly man), Tanzania).*

#### From home to patent medicine vendors

Some respondents reported visiting patent medicine vendors (PMVs, also referred to as chemists) when there was no relief from herbs and drugs used for self-medication at home.

*You have to take enema to wash out all the dirt from the stomach from what you ate. This will give you some relief but if it doesn’t, you have to go to the chemist to get some drugs [FGD Respondent 7 (elderly man), Okpok Ikpa, Nigeria].*

#### From patent medicine vendors (chemists) to health facilities

Health facilities were visited when drugs bought from PMVs did not relieve symptoms or when an illness was exacerbated.

*From the chemist, if that disease does not subside, we can now find way to go to the health centre [FGD Respondent 6 (middle-aged man), Okpok Ikpa, Nigeria].*

*The convenient way is getting medication from the chemist and if the conditions persist, we seek help from the hospitals [FGD Respondent 1, (young man), Kenya].*

#### From health facilities back to patent medicine vendors or traditional healers

After undergoing a consultation with health personnel, some respondents bought medications prescribed by doctors from PMVs due to frequent drug stock-outs in health facilities. There were instances in which respondents were referred back home to take herbs.

*The doctor in the health facility instructed me to buy the drugs from the facility’s pharmacy. But when I went there, it was unavailable. So, I went and bought the drugs from a chemist [FGD Respondent 5, (middle-aged woman), Okpok Ikpa, Nigeria].*

*Some people are referred back to their homes to use native treatment [FGD Respondent 6, (young man), Ikire, Nigeria].*

#### Religion and traditional medicine

A few respondents reported seeking care in outreach programmes organised by churches during which people were tested and given medications. It was a common practice in a few sites for: people to consult their pastors for prayers and receive supernatural healing, pregnant women to seek help from churches where herbs and prayers were prescribed and traditional birth attendants (TBA) to prescribe a combination of traditional medicine and prayers to pregnant women for a safe delivery. The TBAs and spiritual homes sometimes referred cases to the health facilities.

*There are times churches will come with loudspeakers and invite people to come. They (pastors) will give them drugs and test them for about three days and go back again [FGD Respondent 1 (elderly man), Olorunda, Nigeria].*

*When I visit them (sick persons), they say they were prayed for by their pastor so they are well. The challenge is that you cannot force them to go to a hospital [KII with a village leader, Kenya].*

*Pregnant women go there (referring to the church). When you go there, the pastor’s wife will attend to you and give you enema so the baby warms up in your belly. If you need drugs, she advises you on what to take. If it is a good pastor’s wife, she will tell you to go to the hospital [FGD Respondent 3 (middle-aged woman), Okpok Ikpa, Nigeria].*

*There are some people who go to church to give birth but are unable to and they refer them to me. As a Traditional Birth Attendant (TBA), you have to be god-fearing. Whatever is expected of you to help, you have to until she delivers. They live with me in my house. I take them through fasting and prayers until they give birth. I refer difficult cases to the hospital and also refer them for immunization [FGD Respondent 4 (middle-aged woman), Okpok Ikpa, Nigeria].*

### Emerging themes

#### Patronage of patent medicine vendors

Services provided by patent medicine vendors (PMVs) were perceived to be more affordable and accessible than those provided by healthcare providers in health facilities. In addition, they (PMVs) were flexible with instalment payments for fee-for-service and regularly had medications. Furthermore, their clients did not have to wait in long queues to be attended.

*What is the point of going to the hospital when you would be given a prescription to take to the chemist? It is cost effective to take the little money you have to the chemist to buy your drugs [FGD Respondent 3 (young man), Ikire, Nigeria].*

*Another thing I’d like to add that really impressed me about the chemist man (PMV) is that I didn’t have enough money to purchase the drugs, so he asked me to go and bring the money to him later. I was satisfied with his service [FGD Respondent 4 (middle-aged man), Okpok Ikpa, Nigeria].*

*They (doctor) may give you prescriptions and you buy the medicines there (hospital), but they may be selling them (medications) for a higher price than a chemist [IDI with a young woman, Kenya].*

*I go to the chemist to mix drugs for me since the chemist is close to me. They attend to me quickly and give me drugs according to my complaints [FGD Respondent 3 (young woman), Okpok Ikpa, Nigeria].*

#### Quackery by patent medicine vendors (chemists)

Some PMVs, referred to as *Kosongbo* (a Yoruba term which means “run into the bush when you see law enforcement agents”) in western Nigeria, reportedly misdiagnosed their clients and prescribed medications to treat conditions that the medications were not indicated for. A few PMVs were also reported to profiteer from the sale of substandard or expired medicines.

*I had palpitations and asked a Kosongbo to treat me. He gave me moduretic (an antihypertensive medication) which he said I should take twice daily. I almost lost my life in the process and was rushed to the hospital for treatment [FGD Respondent 2 (young woman), Ikire, Nigeria].*

*Again let me also point on the community chemists. Most of the community members are not aware that these are business people who will mostly diagnose one with typhoid. If your situation is complicated, you are diagnosed with typhoid. I recall a case where a son went to a local chemist and was diagnosed with typhoid and was given up to seven jabs (referring to injections). Eventually we went to a public hospital with the situation not improving and he was eventually diagnosed with Tuberculosis [FGD Respondent 5 (young man), Kenya].*

*If it is a bad chemist you patronize, he could sell expired drugs to you which won’t work. You would then start moving from pillar to post (which means to seek help from one place to another) [FGD Respondent 2 (middle-aged man), Okpok Ikpa, Nigeria].*

## Discussion

Access to primary healthcare was limited and, where available, its quality was perceived to be unacceptably poor in public facilities, while costs of care in private facilities was unaffordable in the study sites in East and West Africa. Use of health insurance was considered to be an enabling factor in utilising healthcare in East Africa, while the use of herbal and traditional medicine was associated with delayed utilisation of modern healthcare in Nigeria. These findings are corroborated in other studies which reported health service utilisation factors such as out-of-pocket expenditures; inability to pay premiums; high costs of care, especially in private facilities; physical distance from healthcare facilities; staff shortage and lack of confidence in health insurance schemes were barriers to accessing healthcare [3–5]. In addition to these, our participants also identified unprofessional behaviour of doctors and nurses; unavailability of doctors leading to long waiting time; drug stock-outs; lack of medical equipment and interrupted electricity supply, especially in Nigeria, as other major barriers and quality issues. Many of these factors have previously been reported elsewhere [15, 16, 27].

We observed that herbal enemas are frequently used as the first line of treatment by getting rid of ‘dirt’ in the body, especially in Nigeria. Although plant species used for rectal insertions in this study could not be verified, a study in West Africa reported herbs were prepared from a voucher herbarium plant and administered as enemas to treat a variety of illnesses [28]. However, frequent rectal application of these herbal medicines were reported to be associated with toxicity caused by harmful ingredients, mechanical injury and infections [28]. As reported in a systematic review [29], cultural beliefs around the use of traditional medicines have been identified as key barriers in seeking emergency obstetric care. In some studies, pregnant women preferred the TBAs who administered traditional medicines [30–32]. These practices constitute a barrier to care because of the expectation that obstetric complications would resolve without medical interventions [33]. Furthermore, in this study, pastors’ wives admitted pregnant women in their religious homes and administered prayers and enemas to improve pregnancy outcomes. The role of religion and religious organizations in providing care in resource-limited settings cannot be overemphasized and has been widely discussed in the literature [34–36].

The HB Model [22] explains the complex health-seeking behaviour of participants in this study. For instance, participants’ beliefs about their health problems, perceived benefits of action and barriers to action explain their engagement (or lack of engagement) with the health system. The fact that health facilities were not usually visited as first line of action during illness episodes was due to concerns related to the quality of care: long waiting time, lack of equipment, drug stock-outs and unprofessional conduct of doctors and nurses. These have previously been reported elsewhere [27, 37]. These facility barriers could be the rationale for utilisation of PMVs despite their inappropriate medication dispensing practice, which remains a threat to life and well-being of patients [38]. Respondents considered a visit to a PMV as a better alternative to a facility visit because of perceived benefits such as availability, acceptability, accessibility, affordability (The four “As” of Access to care) and shorter waiting time, with a potential to pay for medicines in instalment [12].

The findings of this study must be interpreted in the light of the following limitations: the design of this study examines the phenomena of access to and quality of care occurring in a bounded context of healthcare facilities which includes non-conventional institutions such as traditional and religious homes and patent medicine stores. Explanations of these phenomena were developed through eliciting lived experiences with healthcare. The qualitative method used precludes the establishment of cause and effect relationships as would be established in quantitative research. Next, the study participants were not randomly selected; hence, the study findings may not be generalised to the population from which the participants were purposively selected. Having one co-author code the data could pose a limitation in this study. Imbalances in the number of methods used for data collection across the sites could be a study limitation. Finally, soft barriers in language, travel, access to patients and recruitment were limitations in this study.

Despite these limitations, our design and methodology are well suited for the study because of the robustness of triangulating the data from healthcare users and providers; and other stakeholders. Another strength of this study lies in its ability to identify and describe the contextual factors that contribute to understanding access to and quality of healthcare in the study settings to generate recommendations for policy and practice in LMICs.

As efforts to increase coverage of primary healthcare expand globally, there is an urgent need to better understand patients’ perspectives on healthcare quality, financial, social and cultural barriers to seeking healthcare from accredited professionals. The balance of public versus private healthcare providers and the links between such systems need to be improved to address severely limited resources in primary healthcare in LMICs. Understanding and possibly incorporating traditional medical practices in primary healthcare systems may help reduce potential for fraud and improve satisfaction with primary healthcare services.

## Conclusions

We think that this is the first multi-country study to use a qualitative approach to describe contextual factors in access to and quality of primary healthcare in SSA by triangulating data from healthcare users and providers and other stakeholders. This study found a substantial gap in primary healthcare coverage and quality in the selected communities in rural and urban areas in East and West Africa. For the purpose of generating recommendations for policy and practice in Nigeria, Kenya, Uganda and Tanzania, key contextual factors that contribute to limited access to and quality of primary healthcare include high insurance premiums, unaffordable costs of care, long distance from healthcare facilities, staff shortage unprofessional behaviour of healthcare providers, long waiting time, drug stock-outs, lack of medical equipment and interrupted electricity supply. Future research should focus on evidence-based interventions to improve access to quality primary healthcare in vulnerable populations in these countries.

## Supplementary Information

**Additional file 1.** Background information of the seven communities in East and West Africa.

**Additional file 2.** The qualitative methods used for the study in the study sites in 2018.

**Additional file 3.** Interview guide developed for the study.

## Data Availability

The data that support the findings of this study are available from [the Bernard Lown Scholars Program in Cardiovascular Health, Department of Global Health and Population, Harvard T.H. Chan School of Public Health, Boston, Massachusetts, U.S.A.] but restrictions apply to the availability of these data, which were used under license for the current study, and so are not publicly available. Data are however available from the authors upon reasonable request and with permission of [the Bernard Lown Scholars Program in Cardiovascular Health, Department of Global Health and Population, Harvard T.H. Chan School of Public Health, Boston, Massachusetts, U.S.A.].

## Abbreviations

FGD: Focus Group Discussion
HB: Health Belief
HCU: Health Care Utilisation
HIV: Human Immunodeficiency Virus
IDI: In-Depth Interview
KII: Key Informant Interview
LMICs: Low- and Middle-Income Countries
PHC: Primary Health Care
PMV: Patent Medicine Vendor
SDGs: Sustainable Development Goals
SSA: Sub-Saharan Africa
TBA: Traditional Birth Attendant
UHC: Universal Health Coverage
WHO: World Health Organization
