# Supplementary material for: A qualitative inquiry of access to and quality of primary healthcare in seven communities in East and West Africa (SevenCEWA): perspectives of stakeholders, healthcare providers and users
Source: BMC Fam Pract. 2021 Feb 25;22:45. doi: 10.1186/s12875-021-01394-z (PMC7908656; doi:10.1186/s12875-021-01394-z)
Supplement: Supplementary file 1 — Additional file 1. Background information of the seven communities in East and West Africa. [file 12875_2021_1394_MOESM1_ESM.docx]

| Country | Community | Details |
| --- | --- | --- |
| Nigeria | Okpok Ikpa | Okpok Ikpa is a rural community in Cross River State, Nigeria with an estimated population of 12,000 people. There is no health facility in Okpok Ikpa. The Department of Community Medicine, University of Calabar, Cross River State has had over a decade-long relationship with the community through provision of community-oriented services and free health outreaches; hence, the rationale for its selection as one of the study sites. |
|  | Ikire | Ikire is a community in Osun State in western Nigeria with a population of about 100,000. It is a peri-urban community with a distance of 30 km to the ancient commercial city of Ibadan, the capital of Oyo State. There is little industrial or commercial development within the town and it is characterised by poor sanitation and low living standards. |
|  | Ogane-Uge | Ogane-Uge is a small vilage in North-Central Nigeria with a population of 3000, mostly farmers. There is only one healthcare center in the area. |
|  | Olorunda Abaa | Olorunda Abaa is a rural community in Oyo State in the western part of Nigeria with an estimated population of 35,000 people. There is only one healthcare center in the area |
| Kenya | Viwandani | Viwandani is an urban slum setting in Nairobi, Kenya with a population of more than 59,000 people in about 28,000 households. The near absence of the public health sector has led to the mushrooming of a vibrant private sector of varied sizes which provide various services. A handful of the private health care providers are credible with qualified health practitioners, but a majority are unqualified and unsupervised by the ministry of health, thereby providing substandard care to the residents. Secondary healthcare is mostly sought in facilities outside of the slum. |
| Uganda | Soroti | Soroti is a small town in eastn Uganda with a population of 50,000, most of whom are serving and retired civil servants. It serves as the commercial hub for Northeastern Uganda, which has a population of about 11 million. The main activities include subsistence agriculture, trading in goods and services. There are currently 8 healthcare centers serving the town’s population. |
| Tanzania | Ukonga | Ukonga is a peri-urban area in the Ilala municipality of Dar es Salaam with a population of about 82,000. Most of the inhabitants are self-employed and don’t have any insurance coverage. There are seven healthcare centers in the area. |
