# Supplementary material for: A qualitative inquiry of access to and quality of primary healthcare in seven communities in East and West Africa (SevenCEWA): perspectives of stakeholders, healthcare providers and users
Source: BMC Fam Pract. 2021 Feb 25;22:45. doi: 10.1186/s12875-021-01394-z (PMC7908656; doi:10.1186/s12875-021-01394-z)
Supplement: Supplementary file 2 — Additional file 2. The qualitative methods used for the study in the study sites in 2018. [file 12875_2021_1394_MOESM2_ESM.docx]

| **Study site** | **FGD** | **KII** | **IDI** |
| --- | --- | --- | --- |
| Okpok Ikpa, Nigeria | 8   - Two FGDs for young people - One FGD for middle-aged women - One FGD for elderly women - One FGD for middle-aged men - One FGD for elderly men - Two FGDs for hypertension patients | - | 1   - Healthcare provider |
| Ikire, Nigeria | 4   - One FGD for young men - One FGD for young women - One FGD for elderly men - One FGD for elderly women | - | - |
| Olorunda, Nigeria | 4   - One FGD for elderly women - One FGD for elderly men - One FGD for young men - One FGD for community leaders and chiefs | 2   - Chairmen of the landlord association - Community chief | - |
| Ogane-Uge, Nigeria | 2   - One FGD for young men and women - One FGD for elderly men and women | - | 1   - Healthcare provider |
| Viwandani, Kenya | 4   - One FGD for young men - One FGD for elderly men - One FGD for young women - One FGD for elderly women | 9   - Health provider - Community health volunteer - Sub-county health management team member - Women group leader - Religious leader - Village chief - Village leader - Community-based organization leader - Youth leader | 7   - Seven selected respondents from the FGDs |
| Soroti, Uganda | - | - | 14   - 10 village leaders - Four healthcare providers |
| Ukonga, Tanzania | 2   - One FGD for elderly men - One FGD for elderly women | - | 2   - One village leader - One councillor |

**Young people (25-44 years of age), middle-aged people (45-60 years of age) and elderly people (60-75 years of age)* (Ref)
