# Supplementary material for: A qualitative inquiry of access to and quality of primary healthcare in seven communities in East and West Africa (SevenCEWA): perspectives of stakeholders, healthcare providers and users
Source: BMC Fam Pract. 2021 Feb 25;22:45. doi: 10.1186/s12875-021-01394-z (PMC7908656; doi:10.1186/s12875-021-01394-z)
Supplement: Supplementary file 3 — Additional file 3. Interview guide developed for the study. [file 12875_2021_1394_MOESM3_ESM.doc]

**INTERVIEW GUIDE DEVELOPED FOR THE STUDY**

**Themes contained in the interview guide**

**I) Access to primary healthcare**

- Tell us about factors that facilitate access to primary healthcare services in your community
  - Probe to know more about provision of free health services
  - Probe to know if use of health insurance facilitates access to health services
- In your opinion, what could be the barriers to accessing primary healthcare in the health facilities in your communities?
  - Invite participants to discuss barriers to healthcare (e.g. poor infrastructure, attitude of health workers, unavailability of doctors and nurses, lack of equipment and medicines)
  - Probe further for out-of-pocket expenditures
  - Probe further for highs costs of accessing healthcare
  - Probe further for physical distance to health facilities
    - How near or far is the nearest health facility from your houses?
    - Invite participants to discuss issues around distance in kilometer, length of time it takes to visit the nearest health facility, and available modes of transportation to the facility
  - Probe for health personnel factors
- In order to discourage out-of-pocket expenditure at the point of care, would you be willing to pay a monthly premium to access care?
  - Probe to know if they are willing to pay and why
  - Ask how much they are willing to pay
  - For those who are unwilling to pay, probe to know the reasons why they are willing to pay

**II) Quality of primary healthcare**

- Tell us whether you are generally satisfied or dissatisfied with the quality of primary healthcare provided in the health facilities in your community
- What challenges hinder the provision of quality care in the health facilities in your communities?
  - Probe for health personnel factors
  - Probe for client waiting time
  - Probe for availability of essential medicines
  - Probe for availability of equipment
  - Probe for consistent power (electricity) supply

**III) Health-seeking behaviour**

- In general, could you tell us what you do when you feel ill?
  - Prompt to know about self-medication
    - Tell us about your past experiences with self-medication
  - Probe to know about visits to patent medicine vendors
    - Tell us about your past experiences with patent medicine vendors
  - Probe to know about health facility visits
    - Tell us about your past experiences with health service providers
    - Tell us about your past experiences with clients (for service providers)
  - Probe to know about visits to traditional birth attendants and healers
    - What is the role of traditional birth attendants and healers in healthcare delivery?
    - Probe to know the details of their roles.
  - Probe to know about visits to religious homes
    - What is the role of churches in the delivery of health care?
    - Probe to know the details of their roles.
